# Association between thyroid autoimmunity and antinuclear antibody prevalence among pregnant women: a cross-sectional study in Qingdao, China

**DOI:** 10.3389/fendo.2024.1403917

**Published:** 2024-06-14

**Authors:** Mingran Wu, Yanzhen Wan, Lin Zhao, Shan Kang, Guiliang Hao, Mingzhen Guo, Shuai Wang, Jianhong Dong, Jinlian Song

**Affiliations:** Department of Laboratory, Affiliated Women and Children’s Hospital of Qingdao University, Qingdao, China

**Keywords:** thyroperoxidase antibody, thyroglobulin antibody, pregnant woman, antinuclear antibody, Chinese

## Abstract

**Objective:**

To identify the relationship between thyroid autoimmunity and antinuclear antibody (ANA) prevalence in Chinese pregnant women.

**Methods:**

The study involved 1923 first-trimester women who were measured for thyroid stimulating hormone (TSH) level, thyroid autoantibodies (thyroperoxidase antibody [TPOAb] and thyroglobulin antibody [TgAb]) and ANA titer. Social demographic data were collected through standardized questionnaires.

**Results:**

In this study, 23.3% of pregnant women tested positive for TPOAb and 9.9% tested positive for TgAb. Women with a positive ANA were more likely to be TPOAb-positive or TgAb-positive than women with a negative ANA (adjusted odds ratio [AOR] 1.96, 95% confidence interval [CI] 1.47–2.62 for TPOAb [+]; AOR 3.12, 95% CI 2.18–4.48 for TgAb[+]). In addition, ANA titers were closely associated with thyroid autoimmunity. Women with an ANA titer of >1:320 had a significant higher risk of being TPOAb positive or TgAb positive (AOR 4.49, 95% CI 1.48–13.66 for TPOAb [+]; AOR 5.51, 95% CI 1.65–18.49 for TgAb [+]). The higher the ANA titer, the greater the risk of developing thyroid autoimmunity, especially for those with a high ANA titer.

**Conclusions:**

ANA positivity is strongly correlated with thyroid autoimmunity. Further study is warranted to clarify the causal relationship between thyroid autoimmunity and ANA in pregnant women.This research is essential to evaluate and predict the risk of co-existing autoimmune disorders,leading to improved care for pregnancy and neonatal health.

## 1 Introduction

Thyroid autoimmunity can manifest through the presence of thyroid autoantibodies against TPO and/or Tg alone, or in conjunction with thyroid dysfunction. Thyroid autoimmunity is common in women, as supported by several comprehensive epidemiological studies indicating that women are about two to three times more likely to test positive for thyroid autoantibodies than men (1, 2). As per the largest study conducted by the National Health and Nutrition Examination Survey (NHANES) III, it was estimated that 17% of women exhibited TPOAb positivity, and 15.2% of women possessed TgAb positivity (3). Even in women with normal thyroid function, thyroid antibodies can have detrimental effects on pregnancy outcomes. These antibodies are considered independent risk factors associated with adverse pregnancy outcomes, such as miscarriage, preterm birth, and perinatal death. In the United Kingdom, a national study on the prevalence of pre-pregnancy TPOAb positivity among women of reproductive age demonstrated an overall prevalence rate of 9.5% (4). Notably, no difference in prevalence was observed even among populations characterized by recurrent miscarriages or low fertility. Two other comprehensive meta-analyses with large sample sizes found that women who tested positive for TPOAb had a higher risk of preterm birth compared to women without TPOAb (3, 5). These findings were further supported by a 2019 meta-analysis conducted by Korevaar et al., which examined the association between thyroid autoimmunity and preterm birth. The analysis revealed that TPOAb positive pregnant women without overt thyroid disease had a higher risk of preterm birth compared to TPOAb-negative women (6). Furthermore, offspring of mothers who tested positive for TPOAb and TgAb showed a higher perinatal mortality rate that was not influenced by the thyroid hormone status (7). Moreover, thyroid antibodies have also been linked to other neonatal complications. Elevated levels of TPOAb during pregnancy are associated with an increased likelihood of attention deficit/hyperactivity disorder in children, according to a study of more than 3,000 children born to TPOAb-positive mothers (8). Likewise, Brown et al. reported similar findings, revealing a significantly higher prevalence of TPOAb positivity among pregnant women with offspring diagnosed with autism compared to the control group (9).

ANA are important biomarkers of autoimmunity because they are directed against nuclear and cytoplasmic proteins, nucleic acids, and their complexes. These biomarkers are highly prevalent in systemic autoimmune diseases, such as systemic lupus erythematosus (SLE), systemic sclerosis, and mixed connective tissue disease (10, 11). ANA play an important role in the diagnosis and categorization of autoimmune diseases. Consequently, ANA testing is extensively performed in clinical practice.

A systematic review and meta-analysis has demonstrated that thyroid autoimmunity is more prevalent in patients with rheumatism than in the general population (12). Thyroid autoimmunity and autoimmune connective tissue diseases (ACTDs) probably co-exist in some patients. Several studies have shown that thyroid autoimmunity is related to ACTDs (13–15), which include SLE, vitiligo (Vit), Sjogren’s syndrome (SS), chronic autoimmune gastritis (CAG), rheumatoid arthritis (RA), and others. Moreover, it was reported that patients with SLE and scleroderma had a higher prevalence of thyroid autoantibodies (2). Patients with systemic connective tissue diseases also showed a higher incidence of thyroid autoimmunity compared to the general population. In fact, autoimmune diseases are common in women and are related to sex hormones such as estrogen and thyroid autoimmunity (16), necessitating the implementation of countermeasures.

Nevertheless, there remains a lack of relevant data on the correlation between thyroid autoimmunity and ANA positivity among pregnant women. Additionally, studies investigating the correlation between demographic factors and thyroid autoimmunity during pregnancy are scarce. Therefore, the present study is aimed to gather essential data on the prevalence of thyroid autoimmunity in pregnant women in Qingdao, China, and to assess the relationship between thyroid autoimmunity and ANA prevalence during pregnancy.

## 2 Materials and methods

### 2.1 Study design and participants

This was a cross-sectional study. Pregnant women aged 19–50 years and at 8–13 weeks of gestation, who underwent health examinations at Qingdao Women and Children’s Hospital affiliated to Qingdao University from March 2023 to December 2023 were included in this study. Pregnant women who exhibited specific high-risk factors for thyroid disorders were not included in the study. These factors included hereditary conditions and chronic illnesses like hypertension, diabetes, and anemia (with hemoglobin levels below 110 g/L), a personal or family history of thyroid diseases, and the use of medication that may impact thyroid function. Pregnant women with missing data on key variables were also excluded. Finally, 1923 eligible participants were successfully included in the study. The Ethics Committee of Qingdao Women and Children’s Hospital affiliated to Qingdao University approved this study (Approval Number: QFELL-KY-2023–03). Following a comprehensive elucidation of the study’s objectives, each participant provided informed consent. For further study, excess serum samples were also collected.

### 2.2 Data collection

In-depth data pertaining to demographic attributes, living conditions, educational background, parity, alcohol consumption, tobacco use, and occupational physical activity were collected through a standardized questionnaire. Because self-reported age was not normally distributed, the subjects were divided into three age groups:<30, 30 to 34, and ≥35 (17). Living conditions were divided into three categories based on the timing of renovation of the pregnant women’s living environment: renovated within 0–6 months, renovated within 6–12 months, and not renovated for more than 12 months. Pre-pregnancy body mass index (BMI) of the subjects was calculated using self-reported height and weight values before conception. According to the classification set forth by the World Health Organization (WHO), BMI is categorized into four groups: low BMI (BMI < 18.5 kg/m²), normal range (BMI: 18.5–24.9 kg/m²), overweight (BMI: 25.0–29.9 kg/m²), and obese (BMI ≥30.0 kg/m²) (18). Self-reported educational levels were categorized into four tiers: middle school or below, high school, university, and postgraduate. Occupational physical activity was divided into three levels (1): sedentary (involving primarily desk-based work, such as secretarial positions), (2) moderate (involving work that needs to stand and walk regularly, such as sales assistants and craftsmen), and (3) vigorous (involving tasks like walking, lifting, and arduous physical labor, such as industrial or agricultural workers) (19). As for parity, pregnant women were categorized into primipara and multipara (20). Folic acid supplements were classified as multivitamin intake, folic acid intake only, or no vitamin intake (21). Maternal smoking, including both active and passive exposure, was assessed during early pregnancy and within 3 months before pregnancy. Active smoking was defined as the daily consumption of at least one cigarette within the specified timeframe, while passive smoking was described as exposure to other individual’s tobacco smoke for a minimum of 15 minutes daily, for more than one day per week during the specified timeframe. Similarly, maternal alcohol consumption was defined as the consumption of at least one standard alcoholic beverage during the specified period.

### 2.3 Establishment of first-trimester specific reference intervals for thyroid hormones

According to the recommendations of the National Academy of Clinical Biochemistry (NACB) (22), we excluded pregnant women with self-reported thyroid dysfunction (e.g., goiter, cancer, hyperthyroidism, or hypothyroidism), those with a clinical or laboratory diagnosis of clear hypothyroidism or hyperthyroidism (abnormal TSH and FT4 values), individuals displaying evidence of autoimmune thyroid diseases (elevated TPOAb and TgAb), women taking or with a history of taking thyroid medications, individuals with a family history of thyroid diseases, and those with incomplete information about thyroid function. We also excluded individuals who had experienced multiple or complicated pregnancies, such as hyperemesis gravidarum, gestational diabetes, hypertension, perinatal infection, and stillbirth. Additionally, we excluded those who had been clinically diagnosed with chronic diseases or autoimmune diseases (such as diabetes, hypertension, asthma, inflammatory bowel disease, tumors, etc.) (23), and those with a history of spontaneous abortion. In total, 141 pregnant women in their first trimester were included to establish the reference interval. When the data follow a Gaussian distribution or are transformed to a normal distribution, reference intervals are computed as follows: mean ± 1.96 × standard deviation (23). In the event of non-attainment of normality, even subsequent to transformation or the exclusion of outliers, a nonparametric methodology is applied for the derivation of reference interval. This involves computation of rank numbers corresponding to the 2.5th and 97.5th percentiles as the lower and upper boundary of the reference interval, respectively.

### 2.4 Laboratory testing

For each specimen, levels of TSH, FT4, FT3, as well as antithyroid antibodies (TPOAb and TgAb), were quantified using the ADVIA Centaur XP Immunoassay System (Siemens Healthcare Diagnostics, ILL 60015–0778, USA). First-trimester reference intervals for TSH, FT4, and FT3 were established in our laboratory. Reference intervals for TPOAb and TgAb were 0–60.0 IU/mL and 0–4.5 IU/mL, respectively, according to the reagent specifications. Hypothyroidism is classified as having a TSH level higher than the upper limit of the pregnancy reference interval established in our laboratory and an FT4 level lower than the lower limit of the pregnancy reference interval established in our laboratory. Subclinical hypothyroidism is classified as having a TSH level above the upper limit of the pregnancy reference interval and an FT4 level falling within the pregnancy-specific reference interval.

The surplus serum specimens were used to detect ANA. ANA titers were determined using an indirect immunofluorescence technique with HEp-2 cells, following a standardized protocol derived from EUROIMMUN in Germany. A titer of ≥ 1:100 was defined as ANA positive. Additionally, staining patterns were documented for all amples that tested positive for ANA.

### 2.5 Statistical analysis

Continuous variables with normal distribution were described as mean ± SD and compared with Student’s t test; non-normal distributed continuous variables were expressed as median with interquartile range (IQR) and compared with the Mann-Whitney U-test. Categorical variables were expressed as numbers and percentages and were compared by the Chi-square test. Logistic regression analysis was performed to evaluate the independent associations of various risk factors with thyroid dysfunction and thyroid autoimmunity, and odds ratios (OR, 95% confidence interval [CI]) were calculated for assessment. Multivariable logistic regression models were employed to analyze the interaction between maternal ANA and early pregnancy thyroid disorders, and ANA titers and specific staining patterns were incorporated into the analysis. A p-value of <0.05 was considered to be statistically significant, and all analyses were performed by SPSS software (SPSS Inc., Chicago, IL, USA).

## 3 Results

### 3.1 First-trimester reference intervals for thyroid hormones

After applying the specified exclusion criteria, 141 women (aged between 20 and 45 years old, with gestational weeks ranging from 4 to 8 weeks) were included to establish the reference intervals. Information about thyroid hormones (TSH, FT4, FT3) is presented in **Table 1**, including the median, and the 2.5th and 97.5th percentiles. The reference intervals for serum TSH, FT3, and FT4 were 0.044–3.236 μIU/mL, 3.988–5.709 pg/mL (2.37–8.02 pmol/L), and 12.710–19.486 pg/mL (12.23–19.69 pmol/L), respectively.

**Table 1:** Reference intervals for TSH, FT3 and FT4 in first trimester of pregnancy women.

	n	2.5th percentile	97.5th percentile	Median	Reference range
TSH (μIU/mL)	141	0.044	3.236	1.70	0.044~3.236
FT3 (pg/mL)	141	3.988	5.709	4.79	3.988~5.709
FT4 (pg/mL)	141	12.710	19.486	15.82	12.710~19.486

TSH, thyroid stimulating hormone; FT3, free triiodothyronine; FT4, free thyroxine.

### 3.2 Participant characteristics and thyroid autoimmunity prevalence.

**Figure 1:**
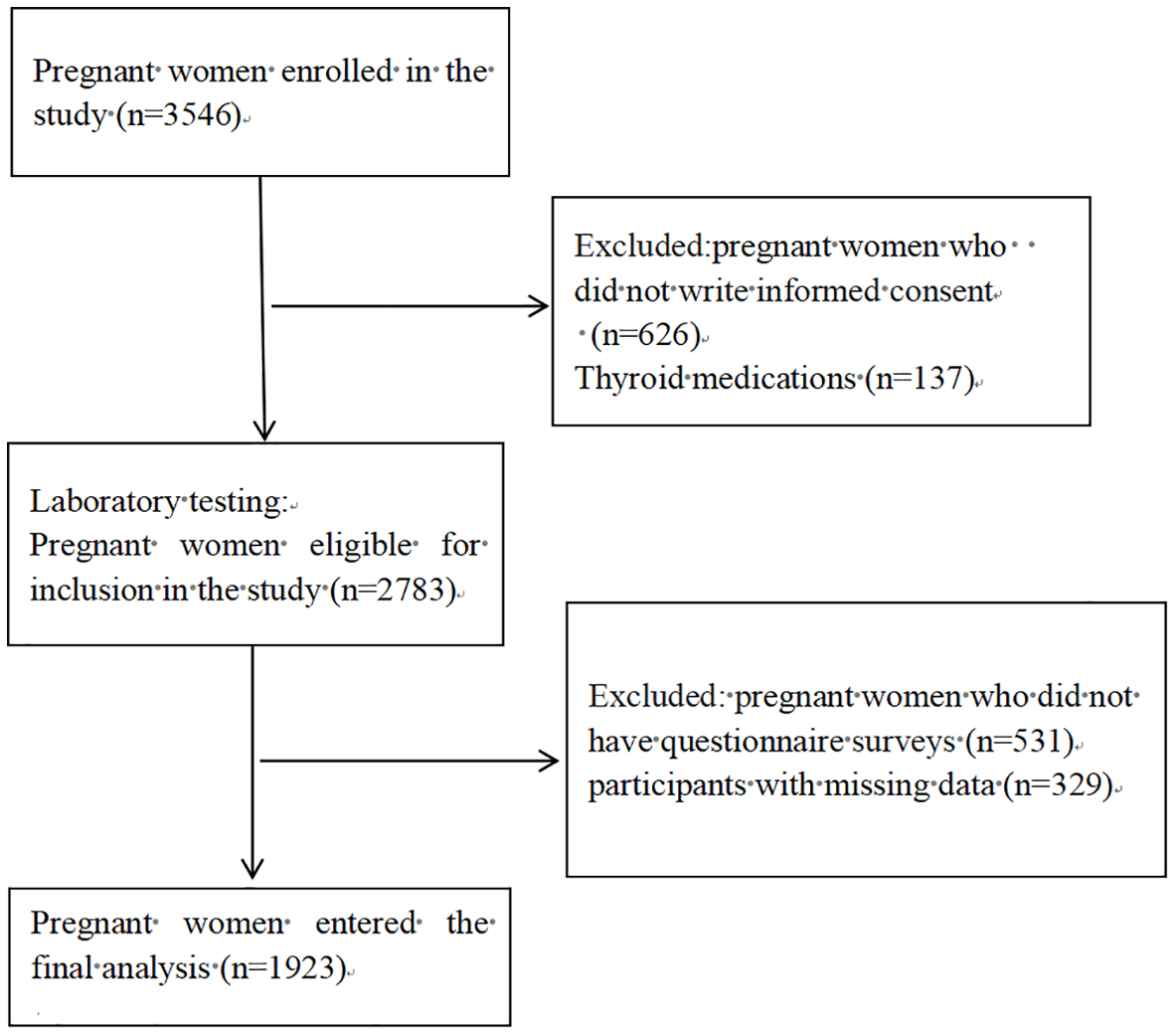
Flowchart illustrating the process of participant recruitment.

We enrolled 3546 women in the first trimester, collected surplus serum samples from 2783 consenting participants. A detailed questionnaire survey was conducted among 2252 pregnant women. Following the exclusion of 329 participants with high risk factors for thyroid disease or with missing data, the final analysis included 1,923 pregnant women in their first trimester (**Figure 1**).

General characteristics of the participants categorized by the status of thyroid autoimmunity are displayed in **Table 2**. The overall incidences of pregnant women positive for TPOAb and TgAb were 23.3% and 9.9%, respectively. The rates of TPOAb and TgAb positivity in pregnant women with euthyroidism were 22.6% and 9.5%, respectively, indicating that most of the women with thyroid autoimmunity had normal thyroid function. For pregnant women aged <30, 30–34, and ≥35, the positive rates of TPOAb were 20.6%, 24.2%, and 25.9%, respectively. The positive rates for TgAb were 8.7%, 9.9%, and 11.5%, respectively. The prevalence of hypothyroidism or subhypothyroidism was 4.2%, 3.0%, and 2.7%, respectively. The proportion of pregnant women with other thyroid function diseases was 8.3%, 14.4% and 16.7%, respectively. Positive TPOAb was significantly associated with age and pre-pregnancy BMI. Hypothyroidism or subhypothyroidism were significantly associated with age and average household income.

**Table 2:** Characteristics of the study population grouped according to thyroid disease.

	Thyroid autoimmunity						Thyroid dysfunction			
	TPOAb		P	TgAb		P	Hypothyroidism or Subhypothyroidism	Other Thyroid Dysfunction Diseases	Healthy pregnant women	P
	+ (n=449)	− (n=1474)		+ (n=191)	− (n=1732)		(n=65)	(n=243)	(n=1615)	
Age group, year			0.085			0.314				<0.01
<30	146 (20.6%)	563 (79.4%)		62 (8.7%)	647 (91.3%)		30 (4.2%)	59 (8.3%)	619 (87.4%)	
30~34	183 (24.2%)	573 (75.8%)		75 (9.9%)	681 (90.1%)		23 (3.0%)	109 (14.4%)	625 (82.6%)	
≥35	115 (25.9%)	329 (74.1%)		51 (11.5%)	393 (88.5%)		12 (2.7%)	74 (16.7%)	358 (80.6%)	
Living condition, month			0.329			0.942				0.317
0–6	362 (22.9%)	1216 (77.1%)		156 (9.9%)	1422 (90.1%)		47 (3.0%)	204 (12.9%)	1327 (84.1%)	
6–12	10 (18.9%)	43 (81.1%)		6 (11.3%)	47 (88.7%)		2 (3.8%)	7 (13.2%)	44 (83.0%)	
>12	74 (26.4%)	206 (73.6%)		28 (10.0%)	252 (90.0%)		15 (5.4%)	31 (11.1%)	234 (83.6%)	
Average household income, n (%)			0.216			0.880				0.016
<100000	30 (23.8%)	96 (76.2%)		14 (11.1%)	112 (88.9%)		3 (2.4%)	23 (18.3%)	100 (79.4%)	
100000–200000	29 (17.8%)	134 (82.2%)		16 (9.8%)	147 (90.2%)		4 (2.5%)	31 (19.0%)	128 (78.5%)	
>200000	388 (23.9%)	1238 (76.1%)		158 (9.7%)	1468 (90.3%)		58 (3.6%)	187 (11.5%)	138 (84.9%)	
Educational level, n (%)			0.296			0.752				0.053
Secondary school or below	30 (23.8%)	96 (76.2%)		14 (11.1%)	112 (88.9%)		3 (2.4%)	23 (18.3%)	100 (79.4%)	
High school	29 (17.8%)	134 (82.2%)		16 (9.8%)	147 (90.2%)		4 (2.5%)	31 (19.0%)	128 (78.5%)	
Junior college College/university	336 (24.2%)	1052 (75.8%)		139 (10.0%)	1249 (90.0%)		50 (3.6%)	157 (11.3%)	1181 (85.1%)	
Postgraduate	52 (21.8%)	186 (78.2%)		19 (8.0%)	219 (92.0%)		8 (3.4%)	30 (12.6%)	200 (84.0%)	
Smoking, n (%)			0.761			0.932				0.235
never	444 (23.3%)	1458 (76.7%)		189 (9.9%)	1713 (90.1%)		64 (3.4%)	243 (12.8%)	1595 (83.9%)	
Smoking	5 (26.3%)	14 (73.7%)		2 (10.5%)	17 (89.5%)		1 (5.3%)	0 (0.00%)	18 (94.7%)	
Passive smoking, n (%)			0.731			0.512				0.440
no	403 (23.5%)	1315 (76.5%)		172 (10.0%)	1546 (90.0%)		56 (3.3%)	212 (12.3%)	1450 (84.4%)	
yes	42 (22.3%)	146 (77.7%)		16 (8.5%)	172 (91.5%)		7 (3.7%)	29 (15.4%)	152 (80.9%)	
Drinking, n (%)			0.298			0.278				0.996
never	444 (23.5%)	1447 (23.5%)		186 (9.8%)	1705 (90.2%)		64 (3.4%)	239 (12.6%)	1588 (84.0%)	
Drinking	5 (15.6%)	27 (84.4%)		5 (15.6%)	27 (83.4%)		1 (3.1%)	4 (12.5%)	27 (84.4%)	
Occupational physical, n (%)			0.753			0.514				0.919
Light	376 (23.3%)	1238 (76.7%)		162 (10.0%0	1452 (90.0%)		55 (3.4%)	202 (12.5%)	1356 (84.1%)	
Moderate	31 (21.7%)	112 (78.3%)		15 (10.5%)	128 (89.5%)		6 (4.2%)	21 (14.7%)	116 (81.1%)	
Active	1920.4%)	74 (79.6%)		6 (6.5%)	87 (93.5%)		3 (3.2%)	11 (11.8%)	79 (84.9%)	
Pre-pregnancy BMI, kg/m^2^			0.011			0.465				0.377
<18.5	24 (13.6%)	153 (86.4%)		12 (6.8%)	165 (93.2)		4 (2.3%)	18 (10.2%)	115 (87.6%)	
18.5–24.9	312 (24.0%)	988 (76.0%)		131 (10.1%)	1169 (89.9%)		48 (3.7%)	163 (12.5%)	1089 (83.8%)	
25.0–29.9	89 (26.0%)	253 (74.0%)		38 (11.1%)	304 (88.9%)		7 (2.0%)	49 (14.3%)	286 (83.6%)	
≥30.0	20 (23.5%)	65 (76.5%)		8 (9.4%)	77 (90.6%)		5 (5.9%)	11 (12.9%)	69 (81.2%)	
Parity, n (%)			0.905			0.212				0.051
primipara	391 (23.3%)	1285 (76.7%)		173 (10.3%)	1503 (89.7%)		58 (3.5%)	203 (12.1%)	1415 (84.4%)	
multipara	50 (23.7%)	161 (76.3%)		16 (7.6%)	195 (92.4%)		6 (2.8%)	38 (18.0%)	167 (79.1%)	
Folic acid supplements, n (%)			0.980			0.542				0.889
No	17 (24.3%)	53 (75.7%)		5 (7.1%)	65 (92.9%)		3 (4.3%)	10 (14.3%)	57 (81.4%)	
Folicacid alone	139 (23.3%)	457 (76.7%)		56 (9.4%)	540 (90.6%)		21 (3.5%)	69 (11.6%)	505 (84.9%)	
Multi-vitamin	292 (23.6%)	945 (76.4%)		130 (10.5%)	1107 (89.5%)		41 (3.3%)	161 (13.0%)	1036 (83.7%)	

TPOAb, thyroperoxidase antibody; TgAb, thyroglobulin antibody; BMI, Body Mass Index.

Other thyroid disorders include hyperthyroidism, subclinical hyperthyroidism, and hypothyroidism.

### 3.3 Association between general characteristics, nutrient-related biomarkers, thyroid dysfunction and thyroid autoimmunity

To further evaluate the association between general characteristics, nutrient-related biomarkers, thyroid dysfunction and thyroid autoimmunity, univariable logistic regression was performed. As shown in **Tables 3**, **4**, TPOAb and TgAb positivity were not significantly associated with educational level, smoking habit, drinking habit, occupational physical activity, parity, folic acid supplements, or serum ferritin, vitamin B12 and urinary iodine concentration/urinary creatinine concentration (UIC/Cr). Remarkably, there was an evident correlation between pre-pregnancy BMI and thyroid autoimmunity during the first trimester of pregnancy. Compared to pregnant women with a pre-pregnancy BMI <18.5, the ORs (95% CI) of TPOAb positivity were 2.013 (1.285–3.153), 2.243 (1.369–3.673), and 1.962 (1.013–3.797) for women with pre-pregnancy BMI of 18.5–24.9, 25.0–29.9, and ≥30.0, respectively. Age was also significantly associated with TPOAb positivity (OR 1.348, 95% CI 1.019–1.783 for women aged ≥35). In addition, the serum TSH level in women with positive TPOAb was significantly higher than that of women with negative TPOAb, so was the TSH level in women with positive TgAb (**Table 3**, P < 0.05). The serum levels of TSH were associated with TPOAb positivity (AOR, 1.21; 95% CI, 1.09–1.34) and TgAb positivity (AOR, 1.35; 95% CI, 1.18–1.54).

**Table 3:** The Relationship between Thyroid Antibodies and Antinuclear Antibodies and Related Biochemical Indicators.

	only TPOAb		P	only TgAb		P
	+	−		+	−	
FERR (ng/ml)	67.86(43.66–112.30)	72.22(43.76–110.00)	>0.05	68.30(42.45–113.50)	72.00(43.82–110.15)	>0.05
ViB12 (ng/ml)	535.00(418.90–675.05)	527.40(417.20–662.20)	>0.05	504.45(397.00–658.00)	532.65(419.50–670.38)	>0.05
UIC/Cr (ug/g)	0.02(0.01–0.03)	0.02(0.01–0.03)	>0.05	0.02(0.01–0.03)	0.02(0.01–0.03)	>0.05
FT3 (pg/mL)	4.62(4.32–5.02)	4.67(4.36–5.02)	>0.05	4.60(4.29–4.91)	4.66(4.36–5.03)	>0.05
FT4 (pg/mL)	15.10(13.83–16.66)	15.08(13.90–16.41)	>0.05	14.99(13.90–16.57)	15.10(13.87–16.45)	>0.05
TSH (μIU/mL)	1.28(0.02–1.99)	1.10(0.61–1.73)	0.005	1.50(0.84–2.23)	1.10(0.06–1.75)	0.005
ANA,n(%)			<0.001	<0.001		
positive	89(20.8%)	183(12.9%)		55(29.7%)	217(13.1%)	
negtive	337(79.1%)	1229(87.0%)		130(70.3%)	1436(86.9%)	
ANA titers,n(%)			<0.001	<0.001		
<100	337(79.3%)	1229(87.0%)		130(70.3%)	1436(86.9%)	
100	68(16.0%)	146(10.3%)		41(22.2%)	173(10.5)	
320	13(3.1%)	30(2.1%)		10(5.4%)	33(2.0%)	
>1:320	7(1.6%)	7(0.5%)		4(2.2%)	10(0.6%)	
ANA staining pattern,n(%)			0.003	<0.001		
others	12(6.3%)	51(2.9%)		12(6.3%)	51(2.9%)	
AC-1	13(2.9%)	15(1.0%)		11(5.8%)	17(1.0%)	
AC-2	25(5.6%)	56(3.8%)		14(7.3%)	67(3.9%)	
AC-3	26(5.8%)	64(4.3%)		17(8.9%)	73(4.2%)	

FERR, ViB12, UIC/Cr, FT3, FT4, TSH Values are median with interquartile range (IQR).

FERR, Ferritin; VitB12, Vitamin B; UIC/Cr, urinary iodine concentration corrected for creatinine; TPOAb, thyroperoxidase antibody; TgAb, thyroglobulin antibody; TSH, thyroid stimulating hormone; FT4, free thyroxine; FT3, free triiodothyronine.

**Table 4:** Logistic regression results for the associations between demographic characteristics and thyroid disease.

	thyroid autoimmunity, OR		Thyroid dysfunction, OR	
	TPOAb (+)	TgAb (+)	Hypothyroidism or subhypothyroidism	Other Thyroid Dysfunction Diseases
Age				
<30	Ref.	Ref.	Ref.	Ref.
30~34	1.232 (0.962~1.576)	1.149 (0.807~1.636)	0.759 (0.4431~1.322)	1.830 (1.308~2.560)
≥35	1.348 (1.019~1.783)	1.354 (0.916~2.002)	0.692 (0.350~1.368)	2.169 (1.504~3.1227)
Living condition				
>12M	Ref.	Ref.	Ref.	Ref.
6–12M	0.647 (0.310~1.354)	1.149 (0.451~2.927)	0.553 (0.304~1.004)	1.160 (0.776~1.736)
0–6M	0.829 (0.620~1.108)	0.987 (0.646~1.509)	0.709 (0.157~3.210)	1.201 (0.498~2.899)
Average household income				
<100000	Ref.	Ref.	Ref.	Ref.
100000–200000	0.693 (0.390~1.229)	0.871 (0,408~1.859)	1.042 (0.228~4.761)	1.053 (0.578~1.918)
>200000	1.003 (0.655~1.535)	0.861 (0.282~1.573)	1.400 (0.431~4.547)	0.589 (0.365~0.950)
Educational level				
Secondary school or below	Ref.	Ref.	Ref.	Ref.
High school	0.693 (0.390~1.229)	0.871 (0.408~1.859)	1.042 (0.228~4.761)	1.053 (0.578~1.918)
Junior college+ College/university	1.022 (0.666~1.1568)	0.890 (0.497~1.594)	1.411 (0.432~4.605)	0.578 (0.357~0.937)
Postgraduate	0.895 (0.536~1.493)	0.694 (0.336~1.436)	1.333 (0.346~5.135)	0.652 (0.360~1.781)
Smoking				
never	Ref.	Ref.	Ref.	Ref.
Smoking	1.173 (0.420~3.274)	1.066 (0.244~4.651)	1.385 (0.182~10.533)	————
Passive smoking				
no	Ref.	Ref.	Ref.	Ref.
yes	0.939 (0.654~1.347)	0.836 (0.489~1.429)	1.192 (0.534~2.663)	1.305 (0.855~1.991)
Drinking				
never	Ref.	Ref.	Ref.	Ref.
Drinking	0.604 (0.231~1.576)	1.698 (0.646~4.461)	0.919 (0.123~6.869)	0.984 (0.341~2.838)
Occupational physical				
Light	Ref.	Ref.	Ref.	Ref.
Moderate	0.911 (0.602~1.379)	1.050 (0.601~1.837)	1.275 (0.538~3.025)	1.215 (0.746~1.979)
Active	0.845 (0.504~1.418)	0.618 (0.266~1.436)	0.936 (0.287~3.059)	0.935 (0.489~1.787)
Pre-pregnancy BMI				
<18.5	Ref.	Ref.	Ref.	Ref.
18.5–24.9	2.013 (1.285~3.153)	1.541 (0.835~2.845)	1.708 (0.607~4.802)	1.289 (0.770~2.157)
25.0–29.9	2.243 (1.369~3.673)	1.719 (0.874~3.379)	0.948 (0.273~3.290)	1.475 (0.831~2.620)
≥30.0	1.962 (1.013~3.797)	1.429 (0.561~3.637)	2.808 (0.732~10.778)	1.373 (0.616~3.061)
Parity				
primipara	Ref.	Ref.	Ref.	Ref.
multipara	1.021 (0.729~`1.430)	0.713 (0.418~1.215)	0.677 (0.373~2.062)	1.586 (1.083~2.323)
Folic acid supplements				
No	Ref.	Ref.	Ref.	Ref.
Folic acid alone	0.948 (0.532~1.691)	1.348 (0.521~3.487)	0.790 (0.229~2.731)	0.779 (0.380~1.596)
Multi-vitamin	0.963 (0.549~1.690)	1.527 (0.604~3.860)	0.752 (0.226~2.502)	0.886 (0.443~1.770)

TPOAb, thyroperoxidase antibody; TgAb, thyroglobulin antibody; OR, odds ratio; Ref, reference.

Other thyroid disorders include hyperthyroidism, subclinical hyperthyroidism, and hypothyroidism.

### 3.4 Association between ANA status and thyroid autoimmunity

Overall, 426 (23.2%) pregnant women with positive TPOAb received ANA test. 20.8% (89/426) of the participants were ANA positive, which was significantly higher than that of those with negative TPOAb (**Table 3**, P < 0.05). Of whom 68 (16.0%) were ANA positive at a titer of 1:100, 13(3.1%) at 1:320, and 7(1.6%) at >1:320. The most common staining pattern was AC-3 (n = 26, 5.8%), followed by AC-2 (n = 25, 5.6%). 185 (10.1%) pregnant women with positive TgAb also received an ANA test. 29.7% (55/185) of the participants were ANA positive, which was drastically higher than that of those with negative TgAb (**Table 3**, P < 0.05). Of whom 41 (22.2%) were ANA positive at a titer of 1:100, 10 (5.4%) at 1:320, and 4 (2.2%) at >1:320. The most common staining pattern was AC-3 (n = 17, 8.9%) followed by AC-2 (n = 14, 7.3%).

**Table 5** displays the relationship between ANA status and thyroid autoimmunity in pregnant women during the first trimester of pregnancy. The presence of ANA was significantly associated with TPOAb positivity (AOR, 1.96; 95% CI, 1.47–2.62) and TgAb positivity (AOR, 3.12; 95% CI, 2.18–4.48). In addition, ANA titers were also associated with thyroid autoimmunity (AOR 4.49, 95% CI 1.48–13.66 between ANA titer >1:320 and TPOAb positivity) (AOR 5.51, 95% CI 1.64–18.49 between ANA titer >1:320 and TgAb positivity). The risk of thyroid autoimmunity gradually increases with the rise in ANA titers, particularly with high ANA titers. Additionally, there was no significant association between different staining patterns of ANA and thyroid autoimmunity.

**Table 5:** Logistic regression results for the associations between ANA and thyroid antibody.

		TPOAb (+)			TGAb (+)		
		n (%)	crude OR (95%CI)	AOR (95%CI)	n (%)	crude OR (95%CI)	AOR (95%CI)
TSH			1.23 (1.11–1.36)	1.21 (1.09–1.34)		1.32 (1.16–1.50)	1.35 (1.18–1.54)
ANA							
negtive (Ref.)		337 (79.3%)	1.00	1.00	130 (70.3%)	1.00	1.00
positive		88 (20.7%)	1.74 (1.32–2.31)	1.96 (1.47–2.62)	55 (29.7%)	2.80 (1.98–3.96)	3.12 (2.18–4.48)
ANA titers							
<1:100 (Ref.)		337 (79.3%)	1.00	1.00	130 (70.3%)	1.00	1.00
1:100		68 (16.0%)	1.70 (1.24–2.32)	1.91 (1.38–2.64)	41 (22.2%)	2.62 (1.78–3.85)	2.95 (1.98–4.40)
1:320		13 (3.1%)	1.58 (0.82–3.06)	1.74 (0.88–3.41)	10 (5.4%)	3.35 (1.61–6.94)	3.43 (1.59–7.41)
>1:320		7 (1.6%)	3.65 (1.27–10.47)	4.49 (1.48–13.66)	4 (2.2%)	4.42 (1.37–14.28)	5.51 (1.64–18.49)
ANA staining pattern							
others (Ref.)		19 (22.9%)	1.00	1.00	12 (22.2%)	1.00	1.00
AC-1		13 (15.7%)	1.53 (0.88–2.65)	2.022 (0.77–5.34)	11 (20.4%)	2.62 (1.36–5.03)	2.69 (0.93–7.77)
AC-2		25 (30.0%)	3.07 (1.45–6.50)	1.15 (0.54–2.45)	14 (25.9%)	7.19 (3.30–15.68)	0.97 (0.39–2.43)
AC-3		26 (31.3%)	1.58 (0.97–2.57)	0.79 (0.37–1.70)	17 (31.5%)	2.32 (1.27–4.24)	0.73 (0.30–1.78)

Adjusted for age, education, income level, smoking, drinking, parity, BMI.

ANA, antinuclear antibody; TPOAb, thyroperoxidase antibody; TgAb, thyroglobulin antibody; AOR, adjusted odds ratio; CI, confidence interval.

## 4 Discussion

Our study revealed a notable prevalence of TPOAb and TgAb positivity among pregnant women during the first trimester in Qingdao, China. Additionally, we observed a significant correlation between thyroid autoimmunity and the presence of ANA, higher pre-pregnancy BMI, and advanced maternal age.

Our findings show that the overall prevalences of TPOAb and TgAb positivity in pregnant women at the first trimester were 23.3% and 9.2%, respectively. These rates were higher than those reported in some studies conducted on the general population of women who are pregnant (24, 25). The prevalence of thyroid autoimmunity differs in different races, and iodine intake is closely associated with thyroid autoimmunity. It was reported that urinary iodine concentration was in a U-shape association with the positive rates of thyroid autoantibodies in pregnant women. In addition, several studies have observed a decrease in both the prevalence and titers of thyroid autoantibodies while pregnant. Hence, variations in participant demographics, pregnancy progression, and iodine intake levels could account for the disparities in reported thyroid antibody prevalence. Additionally, discrepancies may arise from variations in assay methodologies and cutoff thresholds across different studies.

Thyroid autoimmunity presents several risk factors, such as a familial history of thyroid autoimmune disorders, older age, excessive or insufficient iodine intake, among others (3, 25). Our findings indicate that the prevalence of TPOAb positivity in pregnant women aged <30, 30–34, and ≥35 was 20.6%, 24.2%, and 25.9%, respectively. The prevalence of TgAb positivity was 8.7%, 9.9%, and 11.5%, respectively. TPOAb positivity was associated with older age, which was consistent with the findings of Hollowell JG et al. They found that the positive rates of TPOAb and TgAb increased with age in women in the US (26). Importantly, our study showed that the prevalence of thyroid antibody positivity increased with pre-pregnancy BMI, which was related to TPOAb positivity. This suggests that pre-pregnancy BMI might be a risk factor for thyroid autoimmunity. Furthermore, women with thyroid autoimmunity exhibited a higher baseline TSH level than those without thyroid autoimmunity. TSH concentrations were found to be correlated with TPOAb positivity and TgAb positivity, suggesting that the presence of these antibodies might be an important cause of overt or subclinical hypothyroidism.

Twenty percent of patients with positive anti-thyroid autoantibodies also had positive ANA, according to the study by Siriwsrdhane et al. (27). In the study bySegni et al. (28), 86 out of the 93 children tested positive for autoimmune thyroiditis had positive ANA results, accounting 70% of the cases. According to our findings, women who tested positive for TgAb and TPOAb had ANA prevalence rates of 20.8% and 29.7%, respectively. To establish the accurate prevalence of ANA in autoimmune thyroid disorders, further research is essential. This may involve exploring factors such as ethnicity, age, gender, thyroid function, medication, and specific thyroid conditions. Our study initially revealed significant ANA positivity in Chinese pregnant women with TPOAb or TgAb positivity, aligning with prior research. The strength of this study lies in its extensive prospective cohort, enabling an investigation that covers Qingdao’s entire pregnant population. This ensures a robust study design and a high response rate. We further conducted a detailed examination of ANA patterns and titers. Our study found that ANA positivity was significantly associated with thyroid autoimmunity, irrespective of ANA titers and ANA patterns. Exposure to different ANA titers was also associated with TPOAb and TgAb positivity. Our results showed that the risk of thyroid autoimmunity gradually increase with the gradual increasing of ANA titers, especially with high titers of ANA. ANA positivity is a risk factor for thyroid autoimmunity. However, as shown by G. Lanzolla et al., being ANA positive protected against the development of Graves’ disease and reduced its severity (29). The study of ANA patterns is of tremendous interest in the fields of immunology and rheumatology since distinct patterns may have diverse clinical, diagnostic, and prognostic implications. Our results showed that AC-3 and AC-2 were the most commonly observed ANA patterns in pregnant women with TPOAb and TgAb positivity, but the correlation was not statistically significant. AC-2 was also the most common in otherwise healthy subjects (30).

There are some limitations to our study. Initially, participants were recruited from a single women’s and children’s health care center. Our findings might not be representative of those at other institutions around the nation. Consequently, one should proceed with caution when extrapolating these findings. Secondly, there might have been recall bias because pregnant women provided the epidemiological data. Thirdly, this research is an observational cross-sectional baseline study. However, the study is part of our prospective cohort, which allows for an investigation involving Qingdao’s pregnant population, ensuring a robust study design and a high response rate. We also monitored the medications and management given to expectant mothers who tested positive for TgAb or TPOAb. We will keep track of these women, monitor the outcomes of their pregnancies, and track the development of their offspring in future research.

Despite several limitations, our findings indicate a strong association between thyroid autoimmunity, specifically TPOAb positivity or TgAb positivity, and the occurrence of ANA positivity in pregnant women. This study lays a valuable foundation for further research to clarify the causal relationship between thyroid autoimmunity and ANA positivity in pregnant women, which will help to evaluate and predict the risk of co-existing autoimmune disorders to enhance pregnancy and neonatal health care.

## Data availability statement

The raw data supporting the conclusions of this article will be made available by the authors, without undue reservation.

## Ethics statement

The studies involving humans were approved by Ethics Committee of Qingdao Women and Children’s Hospital. The studies were conducted in accordance with the local legislation and institutional requirements. The participants provided their written informed consent to participate in this study.

## Author contributions

MW: Data curation, Methodology, Writing – original draft, Writing – review & editing. YW: Formal Analysis, Methodology, Writing – review & editing. LZ: Methodology, Supervision, Writing – review & editing. SK: Data curation, Writing – review & editing. GH: Writing – review & editing. MG: Writing – review & editing. SW: Writing – review & editing. JD: Writing – review & editing. JS: Conceptualization, Resources, Writing – review & editing.

## References

[1] Ticconi C Pietropolli A Borelli B Bruno V Piccione E Bernardini S. Antinuclear autoantibodies and pregnancy outcome in women with unexplained recurrent miscarriage. Am J Reprod Immunol. (2016) 76:396–9. doi: 10.1111/aji.12560 27616598

[2] Chen S Yang G Wu P Sun Y Dai F He Y. Antinuclear antibodies positivity is a risk factor of recurrent pregnancy loss: A meta-analysis. Semin Arthritis Rheum. (2020) 50:534–43. doi: 10.1016/j.semarthrit.2020.03.016 32442739

[3] Hollowell JG Staehling NW Flanders WD Hannon WH Gunter EW Spencer CA. Serum Tsh, T(4), and thyroid antibodies in the United States population (1988 to 1994): national health and nutrition examination survey (Nhanes iii). J Clin Endocrinol Metab. (2002) 87:489–99. doi: 10.1210/jcem.87.2.8182 11836274

[4] Sakthiswary R Rajalingam S Norazman MR Hussein H. Antinuclear antibodies predict a higher number of pregnancy loss in unexplained recurrent pregnancy loss. Clin Ter. (2015) 166:e98–101. doi: 10.7417/CT.2015.1827 25945451

[5] Kiuttu J Hartikainen AL Makitalo R Ruuska P. The outcome of pregnancy in antinuclear antibody-positive women. Gynecol Obstet Invest. (1994) 37:160–3. doi: 10.1159/000292548 8005543

[6] Kasagi K Takahashi N Inoue G Honda T Kawachi Y Izumi Y. Thyroid function in Japanese adults as assessed by a general health checkup system in relation with thyroid-related antibodies and other clinical parameters. Thyroid. (2009) 19:937–44. doi: 10.1089/thy.2009.0205 19678737

[7] Tanska K Gietka-Czernel M Glinicki P Kozakowski J. Thyroid autoimmunity and its negative impact on female fertility and maternal pregnancy outcomes. Front Endocrinol (Lausanne). (2022) 13:1049665. doi: 10.3389/fendo.2022.1049665 36714589 PMC9874701

[8] Hansen PS Brix TH Iachine I Kyvik KO Hegedus L. The relative importance of genetic and environmental effects for the early stages of thyroid autoimmunity: A study of healthy Danish twins. Eur J Endocrinol. (2006) 154:29–38. doi: 10.1530/eje.1.02060 16381988

[9] Dhillon-Smith RK Tobias A Smith PP Middleton LJ Sunner KK Baker K. The prevalence of thyroid dysfunction and autoimmunity in women with history of miscarriage or subfertility. J Clin Endocrinol Metab. (2020) 105:2667–77. doi: 10.1210/clinem/dgaa302 32593174

[10] Fukushige M Lu X Satoh M Oda M Ohba T Katoh T. Association between antinuclear antibody positivity and chemical exposure among pregnant Japanese women: A cross-sectional study based on the Japan environment and children's study. Int J Hyg Environ Health. (2023) 248:114094. doi: 10.1016/j.ijheh.2022.114094 36610096

[11] Deroux A Dumestre-Perard C Dunand-Faure C Bouillet L Hoffmann P. Female infertility and serum auto-antibodies: A systematic review. Clin Rev Allergy Immunol. (2017) 53:78–86. doi: 10.1007/s12016-016-8586-z 27628237

[12] Springer D Jiskra J Limanova Z Zima T Potlukova E. Thyroid in pregnancy: from physiology to screening. Crit Rev Clin Lab Sci. (2017) 54:102–16. doi: 10.1080/10408363.2016.1269309 28102101

[13] Szyper-Kravitz M Marai I Shoenfeld Y. Coexistence of thyroid autoimmunity with other autoimmune diseases: friend or foe? Additional aspects on the mosaic of autoimmunity. Autoimmunity. (2005) 38:247–55. doi: 10.1080/08916930500050194 16126513

[14] Fallahi P Ferrari SM Ruffilli I Elia G Biricotti M Vita R. The association of other autoimmune diseases in patients with autoimmune thyroiditis: review of the literature and report of a large series of patients. Autoimmun Rev. (2016) 15:1125–8. doi: 10.1016/j.autrev.2016.09.009 27639841

[15] Posselt RT Coelho VN Pigozzo DC Guerrer MI Fagundes MDC Nisihara R. Prevalence of thyroid autoantibodies in patients with systematic autoimmune rheumatic diseases. Cross-sectional study. Sao Paulo Med J. (2017) 135:535–40. doi: 10.1590/1516-3180.2017.0089110617 PMC10016010 29267515

[16] Vilas Boas L Bezerra Sobrinho C Rahal D Augusto Capellari C Skare T Nisihara R. Antinuclear antibodies in patients with endometriosis: A cross-sectional study in 94 patients. Hum Immunol. (2022) 83:70–3. doi: 10.1016/j.humimm.2021.10.001 34686383

[17] Li G Wei T Ni W Zhang A Zhang J Xing Y. Incidence and risk factors of gestational diabetes mellitus: A prospective cohort study in Qingdao, China. Front Endocrinol (Lausanne). (2020) 11:636. doi: 10.3389/fendo.2020.00636 33042010 PMC7516372

[18] He X Wang P Wang Z He X Xu D Wang B. Thyroid antibodies and risk of preterm delivery: A meta-analysis of prospective cohort studies. Eur J Endocrinol. (2012) 167:455–64. doi: 10.1530/EJE-12-0379 22826476

[19] Thangaratinam S Tan A Knox E Kilby MD Franklyn J Coomarasamy A. Association between thyroid autoantibodies and miscarriage and preterm birth: meta-analysis of evidence. BMJ. (2011) 342:d2616. doi: 10.1136/bmj.d2616 21558126 PMC3089879

[20] Gupta P Jain M Verma V Gupta NK. The study of prevalence and pattern of thyroid disorder in pregnant women: A prospective study. Cureus. (2021) 13:e16457. doi: 10.7759/cureus.16457 34422486 PMC8369967

[21] Havdahl A Wootton RE Leppert B Riglin L Ask H Tesli M. Associations between pregnancy-related predisposing factors for offspring neurodevelopmental conditions and parental genetic liability to attention-deficit/hyperactivity disorder, autism, and schizophrenia: the Norwegian mother, father and child cohort study (Moba). JAMA Psychiatry. (2022) 79:799–810. doi: 10.1001/jamapsychiatry.2022.1728 35793100 PMC9260642

[22] Consortium on Thyroid and Pregnancy—Study Group on Preterm Birth Korevaar TIM Derakhshan A Taylor PN Meima M Chen L. Association of thyroid function test abnormalities and thyroid autoimmunity with preterm birth: A systematic review and meta-analysis. JAMA. (2019) 322:632–41. doi: 10.1001/jama.2019.10931 PMC6704759 31429897

[23] Karakosta P Chatzi L Bagkeris E Daraki V Alegakis D Castanas E. First- and second-trimester reference intervals for thyroid hormones during pregnancy in "Rhea" Mother-child cohort, Crete, Greece. J Thyroid Res. (2011) 2011:490783. doi: 10.4061/2011/490783 22175032 PMC3235891

[24] Moreno-Reyes R Glinoer D Van Oyen H Vandevijvere S. High prevalence of thyroid disorders in pregnant women in a mildly iodine-deficient country: A population-based study. J Clin Endocrinol Metab. (2013) 98:3694–701. doi: 10.1210/jc.2013-2149 23846819

[25] Shi X Han C Li C Mao J Wang W Xie X. Optimal and safe upper limits of iodine intake for early pregnancy in iodine-sufficient regions: A cross-sectional study of 7190 pregnant women in China. J Clin Endocrinol Metab. (2015) 100:1630–8. doi: 10.1210/jc.2014-3704 25629356

[26] Mannisto T Vaarasmaki M Suvanto E. Pregnancy outcomes in women with thyroid peroxidase antibodies. Obstet Gynecol. (2011) 117:174–5. doi: 10.1097/AOG.0b013e3182040b53 21173661

[27] Siriwardhane T Krishna K Ranganathan V Jayaraman V Wang T Bei K. Exploring systemic autoimmunity in thyroid disease subjects. J Immunol Res. (2018) 2018:6895146. doi: 10.1155/2018/6895146 30911555 PMC6399525

[28] Segni M Pucarelli I Truglia S Turriziani I Serafinelli C Conti F. High prevalence of antinuclear antibodies in children with thyroid autoimmunity. J Immunol Res. (2014) 2014:150239. doi: 10.1155/2014/150239 24741574 PMC3987791

[29] Lanzolla G Puccinelli L Giudetti M Comi S Menconi F Maglionico MN. Anti-nuclear autoantibodies in graves' Disease and Graves' Orbitopathy. J Endocrinol Invest. (2023) 46:337–44. doi: 10.1007/s40618-022-01906-3 PMC9859920 36030301

[30] Damoiseaux J Andrade LEC Carballo OG Conrad K Francescantonio PLC Fritzler MJ. Clinical relevance of Hep-2 indirect immunofluorescent patterns: the international consensus on ana patterns (Icap) perspective. Ann Rheum Dis. (2019) 78:879–89. doi: 10.1136/annrheumdis-2018-214436 PMC6585284 30862649

